# Prediction of Individual Gas Yields of Supercritical Water Gasification of Lignocellulosic Biomass by Machine Learning Models

**DOI:** 10.3390/molecules29102337

**Published:** 2024-05-16

**Authors:** Kapil Khandelwal, Ajay K. Dalai

**Affiliations:** Department of Chemical and Biological Engineering, University of Saskatchewan, Saskatoon, SK S7N 5A9, Canada; kak368@usask.ca

**Keywords:** machine learning, artificial intelligence, biofuel, hydrogen, lignocellulosic biomass, supercritical water gasification

## Abstract

Supercritical water gasification (SCWG) of lignocellulosic biomass is a promising pathway for the production of hydrogen. However, SCWG is a complex thermochemical process, the modeling of which is challenging via conventional methodologies. Therefore, eight machine learning models (linear regression (LR), Gaussian process regression (GPR), artificial neural network (ANN), support vector machine (SVM), decision tree (DT), random forest (RF), extreme gradient boosting (XGB), and categorical boosting regressor (CatBoost)) with particle swarm optimization (PSO) and a genetic algorithm (GA) optimizer were developed and evaluated for prediction of H_2_, CO, CO_2_, and CH_4_ gas yields from SCWG of lignocellulosic biomass. A total of 12 input features of SCWG process conditions (temperature, time, concentration, pressure) and biomass properties (C, H, N, S, VM, moisture, ash, real feed) were utilized for the prediction of gas yields using 166 data points. Among machine learning models, boosting ensemble tree models such as XGB and CatBoost demonstrated the highest power for the prediction of gas yields. PSO-optimized XGB was the best performing model for H_2_ yield with a test R^2^ of 0.84 and PSO-optimized CatBoost was best for prediction of yields of CH_4_, CO, and CO_2_, with test R^2^ values of 0.83, 0.94, and 0.92, respectively. The effectiveness of the PSO optimizer in improving the prediction ability of the unoptimized machine learning model was higher compared to the GA optimizer for all gas yields. Feature analysis using Shapley additive explanation (SHAP) based on best performing models showed that (21.93%) temperature, (24.85%) C, (16.93%) ash, and (29.73%) C were the most dominant features for the prediction of H_2_, CH_4_, CO, and CO_2_ gas yields, respectively. Even though temperature was the most dominant feature, the cumulative feature importance of biomass characteristics variables (C, H, N, S, VM, moisture, ash, real feed) as a group was higher than that of the SCWG process condition variables (temperature, time, concentration, pressure) for the prediction of all gas yields. SHAP two-way analysis confirmed the strong interactive behavior of input features on the prediction of gas yields.

## 1. Introduction

Ever-increasing urbanization, modernization, and industrialization of human society have led to an exponential rise in energy consumption. Worldwide primary energy consumption reached to 604 exajoules in 2022, which is a 2.1% rise from the 2021 level even after the slowdown due to COVID-19 [1]. Nearly 494 exajoules of this energy demand is fulfilled by fossil fuel sources, which amounts to 81.8% of total energy consumption [2]. Consumption of non-renewable fuel sources not only leads to fuel scarcity but also results in environmental issues [3]. There is a pressing need of human society to reduce this dependency on fossil fuels and shift to alternative renewable fuel sources. 

Lignocellulosic biomass is easily available and abundant in nature, making it a cost-effective, renewable, and sustainable source of energy generation [4]. Use of lignocellulosic biomass as a fuel source will also democratize the access to energy and improve the socio-economics of countries that do not have reservoirs of fossil fuels [5]. At the recent COP 28 summit, 130 counties participated in the Global Renewables and Energy Efficiency Programs aimed to accelerate the clean energy transition by tripling the worldwide renewable energy generation capacity to 11,000 GW by 2030 and rapidly improving the efficiency [6]. Sixty-six counties also set a target to reduce the emissions by 68% by 2050 compared to 2022 levels [7]. Sustainable production of biofuels from renewable lignocellulosic biomass has the potential to not only aid the achievement of the net zero scenario, but also reduce our dependency on non-renewable fossil fuel sources. 

However, raw lignocellulosic biomass contains a high amount of moisture, which requires pre-treatment for production of biofuels by conventional thermochemical processes [8]. This reduces the efficiency of the convectional thermochemical processes for processing of lignocellulosic biomass. Gasification of lignocellulosic biomass in the presence of supercritical water (SCW) can efficiently process high-moisture-containing biomass without needing to pre-dry the feedstock [9]. At temperatures ≥ 371 °C and pressures ≥ 22.1 MPa, water exists in its supercritical state, and is used as the reaction medium in the supercritical water gasification (SCWG) process [10]. SCWG of lignocellulosic biomass is a promising process for the production of hydrogen. Hydrogen can be used as a clean source of energy for industry and transportation as it produces water as its only combustion product apart from energy generation. It also finds an industrial use as a reducing agent for steel production, in the hydrodesulfurization process in refineries, and for the production of green chemicals, green ammonia, and methanol [11,12]. 

The SCWG process is a complex thermochemical conversion process involving various competing reaction and complex reaction mechanisms [13]. Furthermore, due to the heterogenous nature of lignocellulosic biomass, the chemical composition of biomass differs between different biomass. Even the composition of biomass may change for the same biomass depending on the source of biomass, difference in crop production, season, and aging of the biomass. Biomass availability is also a serious supply chain issue for biofuel production processes. The availability of biomass varies dramatically depending on season, geographical location, cost, and ease of access. Additionally, industries typically use a mixture of a variety of lignocellulosic biomass, the composition of which varies drastically. Therefore, understanding the interactive influence and effects of the properties of biomass on the gas yields of SCWG is pivotal for its commercialization. These properties of biomass along with SCWG reaction conditions have interactive, complex, and non-linear relationships on the gas yields of the SCWG process. 

However, most of the studies have investigated only the effects of SCWG reaction parameters on the SCWG process. Even though the conventional design of experiments using response surface methodology or single univariate methods utilizes experimental datasets, it can only study the effects of a limited number of input features on target features. The number of experimental runs quickly increases with the increase in the number of input features and the level of these features, making the performance of such experiments infeasible. Moreover, univariate methods can only study the effect of one parameter at a time and do not account for interactive behavior, whereas RSM utilizes linear regression techniques which are prone to overfitting of the data. On the other hand, traditional modeling techniques such as thermodynamic simulation, kinetics, and computational fluid dynamics (CFD) are not capable of capturing these non-linear relationships and utilize assumptions for simplifying the complex differential equations. 

Since the advent of machine learning modeling, which is capable of efficiently solving complex equations and captures these non-linear relationships using experimental data, it is finding its use in various applications such as fraud detection [14], sentimental analysis [15], and recommender systems [16]. Interpretable machine learning models are also being used for thermochemical processes for conversion of biomass into biofuels [17,18,19].

Despite the widespread use of machine learning for investigation of complex processes, the literature on the application of machine learning models to the SCWG process is still scarce. These studies have focused mostly on the prediction of hydrogen yields, while other gas yields of SCWG are not considered; these other gases also constitute a significant portion of SCWG gas yield and entail the mechanism of the SCWG process. Furthermore, attention has been paid only towards the prediction power of machine learning models themselves, and very little discussion has been provided about the interpretability of these machine learning models; previous research also lacks the detailed reasoning of the observed results of the effects of input features on gas yields. Moreover, extra parameters such as reactor type, biomass type such as lignocellulosic or non-lignocellulosic, and nature of feedstock such as real feed or model compounds are not considered or accounted for.

To address these knowledge gaps, in this study, eight machine learning models, namely, linear regression (LR), Gaussian process regression (GPR), artificial neural network (ANN), support vector machine (SVM), decision tree (DT), random forest (RF), extreme gradient boosting (XGB), and categorical boosting regressor (CatBoost) with particle swarm optimization (PSO) and a genetic algorithm (GA) optimizer were developed for hyperparameter tuning of machine learning models for prediction of H_2_, CH_4_ CO, and CO_2_ gas yields of various lignocellulosic biomasses from the SCWG process. Reaction temperature (temperature), reaction time (time), feedstock concentration (concentration), and reaction pressure (pressure) were used as input features for SCWG reaction conditions. For biomass characteristics, ultimate analysis (carbon content (C), hydrogen content (H), nitrogen content (N), and sulfur content (S)), proximate analysis (volatile matter (VM), moisture content (moisture), ash content (ash)), and feed type (real feed) were used. In total, a dataset of 166 datapoints for the prediction of the H_2_ yield and 118 datapoints each for predictions of CO, CO_2_, and CH_4_ gas yields, with no missing values, was developed using literature studies, which utilized similar reactor setups and minimized variation in unaccounted-for parameters. Shapley additive explanation (SHAP) was used for interpretable machine learning to determine the most dominant features in the prediction of gas yields. Furthermore, one-way and two-way SHAP analyses were used to investigate the effects of input features and their interactions on the prediction of gas yields. A detailed discussion on the results of these analyses in the perspective of SCWG is provided with detailed reasoning. 

## 2. Methods and Materials

### 2.1. Data Collection, Preprocessing, and Exploratory Data Analysis (EDA)

Data for the development of machine learning models were reviewed from research articles on SCWG of biomass. Collected data were screened to only include lignocellulosic biomass and batch reactors while filtering out the data points for non-lignocellulosic biomass, and continuous and semi-batch reactors. Studies of only batch reactors were included as most of the studies investigating SCWG of lignocellulosic biomass utilize the batch reactor. This is due to the feed pumping limitation of lignocellulosic biomass slurry in continuous reactors, especially at small research scale reactors. Furthermore, continuous reactors suffer from fouling and scaling with local heat spots, deposition of feedstock, and non-uniform heat transfer, especially for solid biomass. Moreover, the reaction mechanism of degradation of biomass in continuous reactors differs greatly with the degradation mechanism in batch reactors. Hence, to limit and eliminate the effects of the reactor on gas yields to ensure consistency, only batch reactor studies of SCWG of lignocellulosic biomass were considered. The dataset consisted of 28 types of different lignocellulosic biomass collected from 16 research articles, which utilized a stainless steel (SS) 316 batch reactor having nearly similar dimensions and reactor setups, which also minimized the effects of unaccounted-for variables such as reactor material, heating rate, and reactor dimensions. These lignocellulosic biomasses comprise cellulose, xylose, lignin, kraft lignin, soybean straw, flax straw, canola straw, rice straw, cotton stalk, wheat straw, canola hull, canola meal, pinewood, orange peel, aloe vera rind, banana peel, coconut shell, lemon peel, pineapple peel, sugarcane bagasse, timothy grass, horse manure, pinecone, canola hull fuel pellet, canola meal fuel pellet, oat hull fuel pellet, barley straw fuel pellet, and partially burnt wood. 

Individual gas yields (H_2_, CO, CO_2_, and CH_4_) of SCWG of lignocellulosic biomass in batch reactors were predicted. In total, 166 datapoints were used for the prediction of hydrogen yield and 118 datapoints each were used for the predictions of CO, CO_2_, and CH_4_ gas yields, as some of the studies only reported hydrogen yield. SCWG of lignocellulosic biomass is primarily dependent on SCWG process conditions and biomass characteristics. Hence, SCWG reaction parameters and biomass characteristics were used as input variables. Reaction temperature (temperature), feedstock concentration (concentration), reaction time (time), and reaction pressure (pressure) were used as SCWG process condition variables. For biomass characteristics, proximate analysis, ultimate analysis, and type of lignocellulosic biomass were used as input features. Carbon content (C), hydrogen content (H), nitrogen content (N), sulfur content (S) for ultimate analysis; while, ash content (ash), volatile matter (VM), and moisture content (moisture) were used for proximate analysis. A categorical feature (real feed) was used to represent the real feedstock or model compound. A value of 1 was assigned to ‘Real Feed’ for a real lignocellulosic biomass and a value of 0 was assigned for model compounds of lignocellulosic biomass. Fixed carbon (FC) and oxygen content (O) were filtered out to avoid collinearity, as these are indirectly calculated by subtracting other components from 100% of proximate and ultimate analyses of biomass, respectively. 

**Figure 1:**
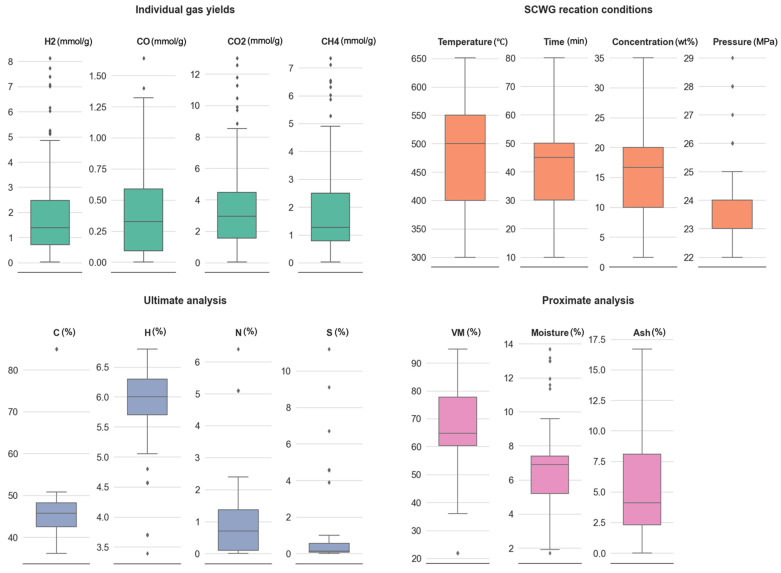
Box plot representation of input and output features.

**Figure 2:**
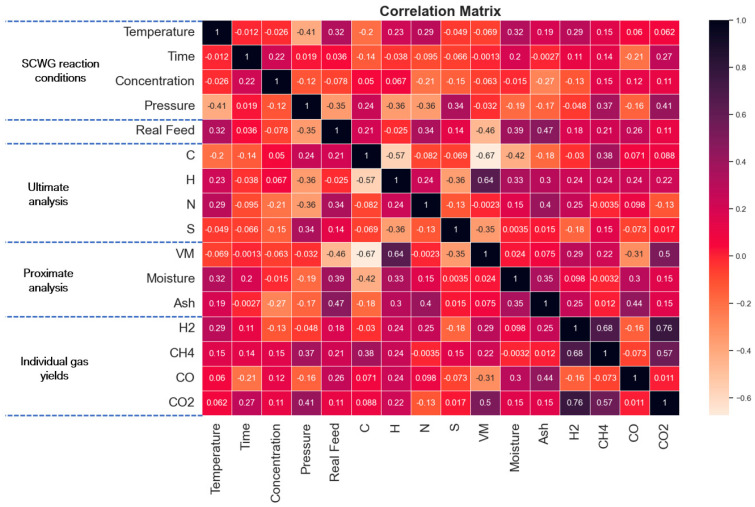
Pearson correlation coefficient (PCC) plot of input and output features.

Exploratory data analysis (EDA) of the developed dataset was conducted for detailed analysis, exploration of the dataset, and identifying the preliminary relationship between input variables and target variables. The distribution of the input dataset is represented via a box plot to analyze the spread of data and identify the outliers (Figure 1). The data were preprocessed to remove outliers and the final dataset consisted of 166 datapoints for hydrogen yield and 118 datapoints each for CO, CO_2_, and CH_4_ gas yields. The relationships between the input variables and gas yields were analyzed using the Pearson correlation coefficient (PCC) matrix (Figure 2). The values of PCC for any two variables vary from +1 to −1. A positive correlation between two features is represented by positive sign and +1 shows a strong positive relation, which indicates that a change in the value of one feature will increase the value of the other feature positively with the same proportion. A negative sign of the PCC value shows a negative relation between two features, meaning an increase in the value of one feature will cause a decrement in the value of the other feature. A PCC value of 0 shows that the two features are not related to each other and a change in the value of either feature will not affect the other feature. PCC values among input features or output features also represent the correlation between a pair of two input variables or a pair of two output variables. 

### 2.2. Machine Learning Model Development and Feature Analysis

Supervised machine learning models such as regression, artificial neural networks, support vector machines, decision trees, and ensemble trees are commonly used machine learning models for biomass conversion processes [17,18,19]. Supervised machine learning utilizes probabilistic and statistical approaches for model building, especially for a structured dataset of a complex process [20]. The performance of these models varies depending on the nature of the relationship between inputs and outputs, number and quality of datapoints, and the complexity of the process. Thus, eight different types of machine learning models (LR, GPR, ANN, SVM, DT, RF, XGB, and CatBoost) were screened for the prediction of gas yields of SCWG. 

The LR machine learning model is simplest, and is based on simple regression techniques. The LR model explains linear relationships quite well; however, it often fails to capture the complex non-linear relationships of the thermochemical processes such as SCWG. Nevertheless, the LR model is used as the baseline to compare and evaluate the performance of other more advanced machine learning algorithms to model the complex non-linear processes. GPR is a non-parametric probabilistic and Bayesian regressor [21]. It is highly flexible and can model complex processes, and the choice of kernel function (covariance function) during the gradient process can control the smoothness, periodicity, and other properties of the GPR model. ANN models are inspired by the functioning and structure of the human brain. A typical ANN is composed of nodes (neurons) organized in layers and each node in one layer connects to nodes in the next layer through weighted links [22]. ANNs are the most commonly used machine learning models for thermochemical processes [17,18,19]. On the other hand, SVMs classify input vectors into distinct categories within a high-dimensional space, and are adept at handling non-linear separations through the use of kernels that project data into higher dimensions where they can be linearly separated [23].

DT, RF, CatBoost, and XGB machine learning models fall under the category of tree-based models. These tree-based models have high accuracy and can handle both categorical and numerical variables having different ranges of values without needing to preprocess the data. The decision tree (DT) model is the simplest and consists of a single tree model based on feature splits on a Boolean condition (True or False) that results in the highest information gain. However, a simple decision tree model is susceptible to overfitting and noise. The RF model improves upon this by creating an ensemble of multiple decision trees in a bagging fashion, each trained on a random subset of the data and features, the results of which are averaged to reduce variance [24], whereas CatBoost and XGB are gradient boosting models that build trees sequentially, where each new tree corrects the errors of the previous trees. The CatBoost model provides an optimized approach and it is a permutation-driven ordered boosting algorithm [25]. XGB is an advanced form of gradient boosting, which offers a performance-oriented architecture that can efficiently handle large-scale data [26]. However, it also requires intricate hyperparameter tuning to achieve optimal performance.

Machine learning models have various parameters for the architect of the models, such as the number of neurons and layers in ANN models, the number of nodes and maximum depth in the case of the decision tree algorithm, or the type of kernel used in the SVM algorithm. The performance of machine learning models is highly dependent on these parameters. For example, a smaller number of neurons in an ANN model can lead to the underfitting of the data, whereas a higher number of neurons can also lead to overfitting and diminishing return, leading to intensive computing. Therefore, optimization of these parameters, commonly referred to as hyperparameters of machine learning models, is needed to ensure the optimal performance of machine learning models while ensuring high efficiency.

In this study, two optimizer algorithms, particle swarm optimization (PSO) and the genetic algorithm (GA), are utilized for hyperparameter tuning of machine learning models. The genetic algorithm (GA) is based on the principle of genetics and natural selection for the survival of the fittest [27]. GA algorithms first start with the initial population of the solutions as analogues to genes or chromosomes, coded as the string of the binary (1 or 0) values. The GA algorithm utilizes genetic methodologies such as selection, mutation, and crossover (recombination) to iteratively update the new generation of the population to adapt to the changing environment, with aim of evolving the population towards the optimal solutions [28]. Crossover and mutations introduce genetic diversity, enabling the algorithm to explore new regions of the solution space. Over various populations, GA converges to provide the population with high-quality genes or solutions.

Particle swarm optimization (PSO), on the other hand, is a heuristic algorithm, based on the collective behavior of animals for feeding [29]. In PSO, each solution is analogous to the food and prey, and is referred to as a particle. A group or swarm of these particles is initiated with a random position in the solution space [24]. These particles then iteratively update their positions to hunt for the best position based on their current position and surrounding points. The aim is to find the global optimum in the solution space. This gives PSO the ability to handle non-linear and multidimensional optimization problems, which is a key asset for optimization of the hyperparameters of the complex machine learning models having multiple parameters to be optimized [30]. Thus, PSO effectively leverages both position and velocity attributes in its search for optimal solutions.

For development of machine learning models, normalization of the clean and preprocessed dataset was achieved using the StandardScaler operation of the sklearn library, which normalizes the dataset around the mean of 0 and standard deviation of 1. This helps to improve the prediction by scaling the input parameters and normalizing their weightings to eliminate the effects of the range of input variables on prediction models. These normalized data were then fed to machine learning models for development. Machine learning models were developed exclusively for each gas yield. The normalized dataset was split into a training dataset and a test dataset. Splitting was performed using a ratio of 80:20 for the training and testing datasets, using train_test_split from the sklearn library of Python. Hyperparameters of each machine learning model for each gas yield were optimized using PSO and GA algorithms, and the effectiveness of the optimizer was compared with unoptimized machine learning models. 

For evaluation of the predictive performance of each developed model, the coefficient of determination (R^2^) and the mean squared error (MSE) were used as evaluation matrices. R^2^ is the measurement of the explainability of the variation in target features (individual gas yields) by the input features [31]. A model with a high value of R^2^ represents high explainability of the target features, indicating a good fit by the developed machine learning model. MSE is the measurement of the square of the difference between the predicted value of the target feature by the machine learning model and the actual value of the target feature in the experimental sample [32]. This represents the absolute measurement of the fit of the dataset by machine learning models. The calculation equations of R^2^ and MSE are provided in Equations (1) and (2).

(1) $${Coefficient\;of\;determination\;(R}^{2})=1-\frac{\sum_{n=1}^{N}{\left(y_{n}-\hat{y_{n}}\right)}^{2}}{\sum_{n=1}^{N}{\left(y_{n}-\bar{y}\right)}^{2}}$$

(2) $$Mean\;squared\;error\;(MSE)=\frac{1}{N}\;{\left(y_{i}-\hat{y_{i}}\right)}^{2}\;$$

Here, *N* represents the total number of data points for output *y*. $y_{n}$ represents the experimental value of output *y* for the *n*^th^ datapoint and $\hat{y_{n}}$ represents the predicted value of output by the machine learning model for the *n*^th^ datapoint, whereas $\bar{y}\;$is the average or mean of all predicted values of output *y* for all datapoints (*N* datapoints). 

After the development and selection of the best performing model for each individual gas yield, interpretation of machine learning is essential to understand how each input feature contributes to and influences the prediction of the model. Shapley additive explanation (SHAP) was used to explain the machine learning models. SHAP is based on game theory, which allocates a contribution value to each input feature [33]. Traditionally, Shapley values were used in corporate games among employees for the distribution of the prizes based on their contribution. In SHAP, each feature is analogous to a player, which, with other players (features), contributes to the prediction of the target feature. The SHAP value is calculated by averaging the marginal contribution of a particular feature across all possible combinations of input features. It helps to develop the relationship and interactive influence of the input feature for each gas yield to understand degradation of complex lignocellulosic biomass in SCWG. It also enables the interpretability of the machine learning model to overcome their black-box nature.

## 3. Results and Discussion

### 3.1. Exploratory Data Analysis (EDA)

EDA of dataset was conducted for preprocessing and assessment of the dataset. Analysis of the distribution and relationship between input features and target variables was conducted to identify the outliers, handling of missing values, and cleaning of the dataset. Missing values for some biomass properties features were filled using the other literature, which had value of missing datapoint for the same feedstock from the same research group. The final preprocessed and cleaned dataset consisted of 166 datapoints for hydrogen yield prediction and 118 datapoints each for CO, CO_2_, and CH_4_ gas yield predictions. 

The distribution of dataset was visualized using the box plot presented in Figure 1. From Figure 1, it can be observed that the ranges of H_2_, CO, CO_2_, and CH_4_ gas yields were (0.02–8.13 mmol/g), (0.00–1.64 mmol/g), (0.03–13.01 mmol/g), and (0.02–7.35 mmol/g), respectively, while their means were 1.84, 0.38, 3.53, and 1.86 mmol/g, respectively. Similarly, ranges of ‘Temperature’, ‘Time’, ‘Concentration’, ‘Pressure’, ‘C’, ‘H’, ‘N’, ‘S’, ‘VM’, ‘Moisture’, and ‘Ash’ input features were (300–651 °C), (10–80 min), (1.64–35.00 wt%), (22–29 MPa), (36.10–85.00%), (3.39–6.80%), (0.00–6.40%), (0.00–11.20%), (21.90–95.00%), (1.71–13.69%), and (0.00–16.70%), respectively. The dataset consisted of a wide range of SCWG process conditions utilized for SCWG of lignocellulosic biomass for improving the scope of the machine learning models. Furthermore, the dataset consisted of both model compounds and real feed for lignocellulosic biomass, incorporating 28 different types of lignocellulosic feedstocks comprising of cellulose, xylose, lignin, kraft lignin, soybean straw, flax straw, canola straw, rice straw, cotton stalk, wheat straw, canola hull, canola meal, pinewood, orange peel, aloe vera rind, banana peel, coconut shell, lemon peel, pineapple peel, sugarcane bagasse, timothy grass, horse manure, pinecone, canola hull fuel pellet, canola meal fuel pellet, oat hull fuel pellet, barley straw fuel pellet, and partially burnt wood. 

Correlations between pairs of two input features or pairs of two output variable, and also between pairs of input features and output variables, are visualized using the PCC correlation matrix (Figure 2). From Figure 2, both temperature and volatile matter (VM) have the highest PCC coefficient of 0.29 for hydrogen yield. This shows that the increment in temperature significantly increases hydrogen yield. It can be also observed that hydrogen content (H) of biomass is highly correlated with VM, with PCC of 0.64. Furthermore, hydrogen content has a high correlation with hydrogen yield, with PCC of 0.24. This shows that the high volatile matter containing biomass usually has high hydrogen content, which enhances the hydrogen yield. Among output parameters, hydrogen is strongly positively correlated with CO_2_ yield. This is due to the fact that, in SCWG, hydrogen is produced mainly via reforming and the water–gas shift reaction since the reforming reaction mainly produces hydrogen with CO, which further undergoes a water–gas shift reaction to produce more hydrogen or is consumed via a methanation reaction to produce methane. Since CO_2_ is also produced via a water–gas shift reaction along with hydrogen; thus, yields of CO_2_ and H_2_ are correlated. 

### 3.2. Evaluation of Machine Learning Models 

LR, GPR, ANN, SVM, DT, RF, XGB, and CatBoost machine learning models were trained on the clean and preprocessed dataset for prediction of gas yields of SCWG of lignocellulosic biomass. Hyperparameters of these machine learning models for each gas yields were optimized using GA and PSO optimizer algorithms. The parameters of GA and PSO optimizer algorithms are presented in Table 1 and Table 2. Hyperparameters and their ranges for optimization of each machine learning model for hydrogen yield are presented in Table 3. The results of the optimized hyperparameters of each machine learning model for hydrogen yield by GA and PSO optimizer algorithms are also presented in Table 3. It can be observed that despite being heuristic optimization algorithms, both GA and PSO algorithms are solved for different optimized hyperparameters. This is due to differences in the search mechanisms of both algorithms to find optimal hyperparameters. Hyperparameters of each machine learning model for the prediction of other gas yields were also optimized using GA and PSO optimizers.

**Figure 3:**
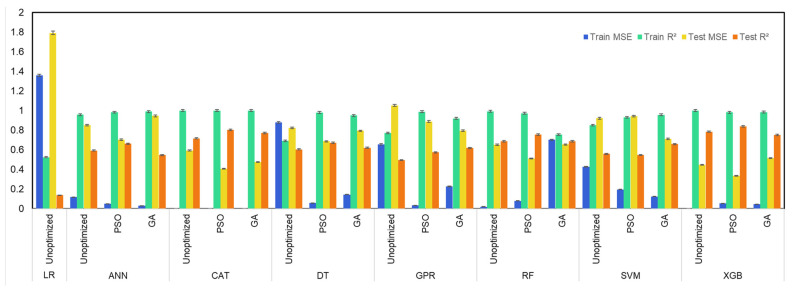
Performance of machine learning models for prediction of gas yields of H_2_ gas yield.

**Figure 4:**
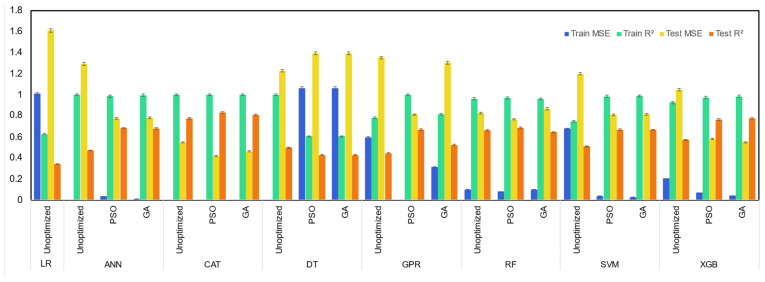
Performance of machine learning models for prediction of gas yields of CH_4_.

**Figure 5:**
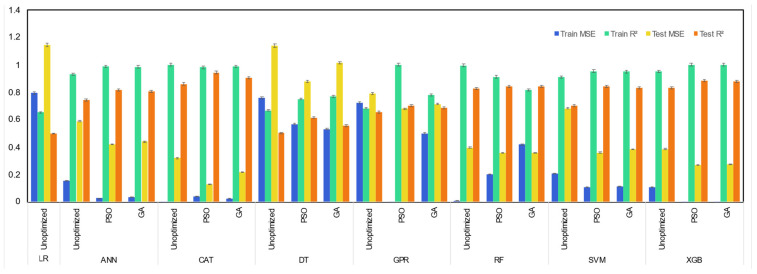
Performance of machine learning models for prediction of gas yields of CO gas yield.

**Figure 6:**
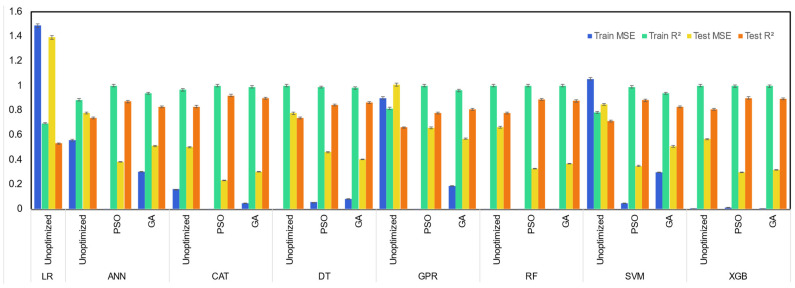
Performance of machine learning models for prediction of gas yields of CO_2_ gas yield.

Unoptimized, GA-, and PSO-optimized machine learning models were compared and evaluated using values of R^2^ and MSE of the respective machine learning models. The results of R^2^ and MSE values during training and testing of the machine learning model are presented in Figure 3, Figure 4, Figure 5 and Figure 6. From Figure 3, it can be observed that for prediction of hydrogen yield, the LR model demonstrated poor performance, with the lowest test R^2^ of just 0.14 and a very high test MSE of 1.79. This is due to the fact that the LR model utilizes a linear regression mechanism during the learning of the machine learning model. It explains the linear relationships between input features and output very well. However, it is not capable of processing complex and non-linear datasets. The poor performance of the LR model signifies the non-linear relationship between input features and hydrogen yield. Similarly, GPR and SVM models also demonstrated moderate performance with a test R^2^ of 0.50 and 0.56, respectively. This is due to the fact that the GPR model is also based on regression analysis, which also suffered due to non-linear relationship of SCWG features. The low performance of the SVM model was also due to non-linearity of the SCWG process, which affects the hyperplane separation and thus affects the performance of the SVM model. 

The ANN model usually shows relatively good performance for thermochemical processes. However, the ANN model demonstrated moderate performance, with its test R^2^ of 0.59. This is due to the fact that simple ANN models are susceptible to overfitting of small datasets, especially those having non-linear complex relationships [34]. This is also confirmed by its relatively high training R^2^ of 0.96 during the training of the model. This highlights the overfitting and biasedness of the ANN model for prediction of the hydrogen yield. A literature study also confirmed the susceptibility of the ANN model for overfitting for prediction of the hydrogen yield. Zhao et al. [35] reported that for prediction of the hydrogen yield from SCWG, ANN and GPR models suffered from overfitting due to the use of a single training process, and these models do not utilize statical averaging or bootstrap sampling compared to ensemble tree models. The SVM model is also susceptible to overfitting due to similar reasons. 

Among all unoptimized machine learning models, tree-based models demonstrated high predictive power for hydrogen yield. The unoptimized XGB model showed a high test R^2^ of 0.78 and low test MSE of 0.45. The XGB model also demonstrated high prediction capabilities during training of the model, with a high training R^2^ of 0.999. This shows the balanced performance of the unoptimized XGB model in both training and testing. The unoptimized CatBoost model also performed well, with its test R^2^ of 0.72 followed by a test R^2^ of 0.69 of the unoptimized RF model. Among tree-based models, the simple DT model demonstrated the lowest test R^2^ of 0.61 compared to ensemble-based tree models. A simple decision tree model is susceptible to overfitting of the dataset, which is minimized in ensemble tree models. Ensemble tree models usually utilize a group of simple decision tree models and either average the prediction of each tree model in the case of the RF model or correct the error of the preceding tree sequentially in the case of the XGB and CatBoost models [36]. This eliminates the biasedness of a single decision tree model and minimizes the overfitting of the dataset by the machine learning model. 

The use of GA and PSO optimizers improved the prediction power of nearly all machine learning models. In general, hyperparameter-tuned machine learning models optimized by the PSO algorithm outperformed the GA algorithm-optimized machine learning models. This is due to the difference in the search mechanism of both algorithms for optimal solutions, which resulted in the different optimized hyperparameters selected by both algorithms. Since the performance of a machine learning model is dictated by its hyperparameters, GA- and PSO-optimized models differ in their prediction capabilities. Among all unoptimized, GA-, and PSO-optimized machine learning models, the PSO-optimized XGB model demonstrated the highest test R^2^ of 0.84 and the lowest test MSE of 0.34. Interestingly, while the use of the PSO optimizer improved the test R^2^ of the XGB model, it also reduced the training R^2^ to 0.98. In contrast, the training R^2^ scores were 0.999 for the unoptimized model and 0.98 for the GA-optimized model. This highlights the effectiveness of the PSO optimizer algorithm in improving the robustness of the model by minimizing the biasedness and overfitting of the model, which resulted in improved performance on the test dataset. PSO-optimized CatBoost also performed well, with its high test R^2^ of 0.80 and low test MSE of 0.41. The order of test R^2^ values among PSO-optimized machine learning models was XGB-PSO > CAT-PSO > RF-PSO > DT-PSO > ANN-PSO > GPR-PSO > SVM-PSO. On the other hand, the order of R^2^ among GA-optimized machine learning models was CAT-GA > XGB-GA > RF-GA > SVM-GA > DT-GA > GPR-GA > ANN-GA. 

For prediction of CH_4_ yield, among unoptimized machine learning models, the CatBoost model showed superior performance, with its high test R^2^ of 0.78 for prediction of methane yield from SCWG of lignocellulosic biomass (Figure 4). The unoptimized RF model also performed well, with its test R^2^ of 0.66. Similar to the prediction of hydrogen yield, the LR machine learning model performed worst among all machine learning models for prediction of methane yield, with its lowest test R^2^ of 0.34 and highest test MSE of 1.60. The performance of machine learning models was improved by the use of PSO and GA optimizer algorithms. However, in general, the improvement in the prediction power of optimized machine learning models from unoptimized machine learning models was higher with the PSO optimizer compared to the GA optimizer. Among all machine learning models, CatBoost models were the top three best performing models, with the PSO-optimized CatBoost machine learning model resulting in the highest test R^2^ of 0.83, followed by 0.81 of the GA-optimized CatBoost model and 0.78 of the unoptimized CatBoost model. GA- and PSO-optimized XGB models also showed comparable performance, with test R^2^ of 0.77 and 0.76. However, LR, GPR, and DT machine learning models demonstrated the worst performance among all machine learning models. 

Similar to the prediction of hydrogen yield and methane yield, CatBoost and XGB were the best performing machine learning models for prediction of CO yield of SCWG of lignocellulosic biomass (Figure 5). Among unoptimized machine learning models, CatBoost models had the highest test R^2^ of 0.86, followed by 0.83 of XGB and 0.83 of the RF model. The extent of improvement in the performance of machine learning models by the PSO optimizer algorithm was also higher compared to the GA optimizer algorithm from unoptimized machine learning models for the prediction of CO yield. The PSO-optimized CatBoost model was the best performing machine learning model for prediction of CO yield, with its highest test R^2^ of 0.94, followed by the GA-optimized CatBoost and PSO-optimized XGB machine learning model. Among all machine learning models, LR and GPR were the worst performing machine learning models. 

The CatBoost model was also able to predict the CO_2_ gas yield of the SCWG process (Figure 6). Among unoptimized machine learning models, the CatBoost model demonstrated high prediction performance, with its test R^2^ of 0.83, followed by test R^2^ of 0.81 of XGB and 0.78 of RF. The PSO optimizer further improved the performance of machine learning models, and the CatBoost-PSO model had the highest test R^2^ of 0.92 followed by 0.90 of XGB-PSO among all machine learning models. Similar to the prediction of other gas yields, the PSO optimizer performed better compared to the GA optimizer in improving the performance of unoptimized machine learning models. SVM, GPR, and LR had the worst performance in the prediction of CO_2_ gas yield. 

Thus, boosting ensemble-based machine learning models such as CatBoost and XGB were clearly the best performing machine learning models for the prediction of gas yields of the SCWG process. This is due to the use of the tabular and structured dataset, for which ensemble tree-based model tends to perform the best [37]. Boosting ensemble tree models have also demonstrated their superior prediction power in other thermochemical processes such as pyrolysis [38], hydrothermal liquefaction [39], and hydrothermal carbonization [40]. Moreover, these boosting models utilize the decision of a group of multiple simple tree models, which learn from the preceding tree specifically for the misclassified instances. This limits overfitting, especially for smaller dataset. Studies also showed that the ensemble boosting algorithms outperform even the deep learning models for a variety of tabular datasets [41]. 

In conclusion, XGB-PSO and CatBoost-PSO models demonstrated the highest prediction power for the yields of H_2_, and CH_4_, CO, and CO_2_, respectively, for SCWG of lignocellulosic biomass. Overall, the effectiveness of the PSO optimizer for hyperparameter tuning of machine learning models was highest compared to the GA optimizer. Due to the superior performance of these machine learning models, XGB-PSO and CatBoost-PSO were selected for further analysis of the prediction of H_2_, and CH_4_, CO, and CO_2_ gas yields, respectively. 

### 3.3. Feature Analysis and Summary Plots

The impact of input features and their relative importance for prediction of gas yields of SCWG process in machine learning models were studied using SHAP analysis. SHAP analysis helps to overcome the black-box nature of machine learning models. SHAP values quantify the contribution of each feature towards the prediction of a machine learning model. The ‘base case’ refers to the model’s prediction using no feature information. Thus, a positive SHAP value indicates that a feature has increased the prediction from the base case, while a negative value indicates a decrease in the prediction power of the machine learning model. A SHAP value of zero suggests the feature has no impact from the base case prediction. This metric offers an intuitive means to interpret complex model predictions. 

**Figure 7:**
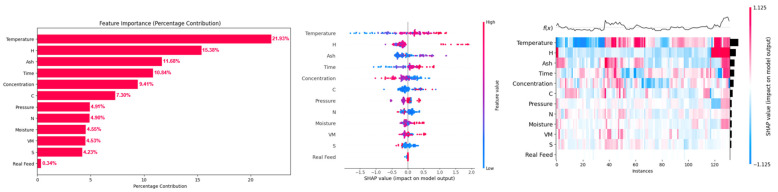
SHAP feature importance, summary plot, and heat map of input features on prediction of H_2_ yield in the PSO-optimized XGB model.

**Figure 8:**
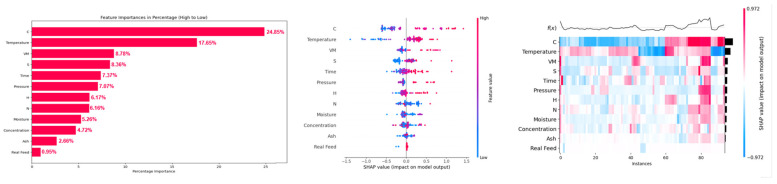
SHAP feature importance, summary plot, and heat map of input features on prediction of CH_4_ yield in the PSO-optimized CatBoost model.

**Figure 9:**
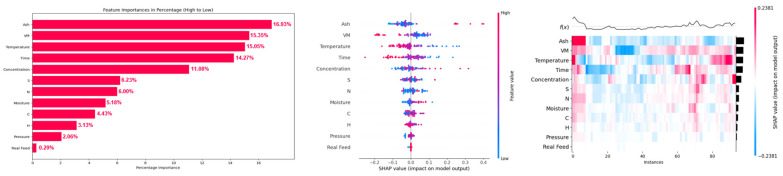
SHAP feature importance, summary plot and heat map of input features on prediction of CO yield in the PSO-optimized CatBoost model.

**Figure 10:**
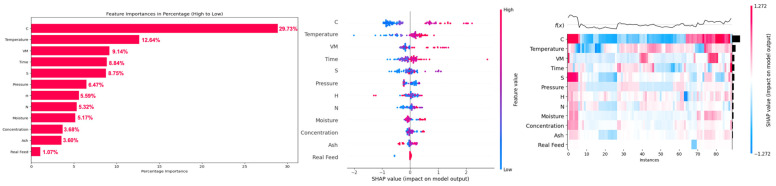
SHAP feature importance, summary plot and heat map of input features on prediction of CO_2_ yield in the PSO-optimized CatBoost model.

Feature importance plots, summary plots, and heat maps of SHAP values of input features for prediction of H_2_, CH_4_, CO, and CO_2_ yields are presented in Figure 7, Figure 8, Figure 9 and Figure 10. From Figure 7, it can be observed that the temperature was the most dominant feature, with feature importance of 21.93%, followed by (15.38%) hydrogen content (H), (11.68%) ash content (ash), (10.84%) time, (9.41%) concentration, and (7.30%) carbon content (C) for prediction of hydrogen yield by the XGB-PSO model. The high feature importance of temperature for hydrogen yield is due to the endothermic nature of reforming, hydrolysis, and water–gas shift reactions, which are favored at high reaction temperatures, enhancing the hydrogen yield [42]. The SHAP summary plot also shows that an increase in the feature value of temperature increased the SHAP values of hydrogen yield and shifted the SHAP value points to right side (more positive). A more detailed analysis of each instance is provided in the heat map of the SHAP value plot for hydrogen yield.

Hydrogen content was the second most dominant feature for prediction of hydrogen yield, which increased hydrogen yield with an increase in the value of hydrogen content. This shows that hydrogen content of biomass contributes to the hydrogen yield, and biomass with higher hydrogen content is recommended to achieve high hydrogen yield. An increase in ash content also improved hydrogen yield. This is due to the presence of alkali and alkaline earth metals (AAEMs) in the ash content of lignocellulosic biomass [43]. These AAEMs have catalytic effects in promoting reforming, hydrolysis, and water–gas shift reactions, which improves the hydrogen yield [44]. However, higher values of ash content also decreased the hydrogen yield, as indicated in the summary plot. Thus, only the optimum amount of ash content in biomass is beneficial for hydrogen yield. Different nature of components of ash content should also be considered as some of these components in ash content may be less or more active in their catalytic activity for SCWG. For example, silica (SiO_2_) present in ash content has very little catalytic activity compared to highly active potassium (K) for the SCWG reaction. 

Increase in reaction time (time) also increased the hydrogen yield since longer reaction time allows sufficient time for hydrolysis and reforming reactions to take place that produce hydrogen and CO, enabling the water–gas shift reaction of CO in excess water for production of more hydrogen [45]. Therefore, higher reaction time is beneficial for high hydrogen yield. However, an increase in feedstock concentration decreased the hydrogen yield since hydrogen is also produced from supercritical water (SCW), which acts as a reactant in reforming, hydrolysis, and water–gas shift reactions. Additionally, feedstock concentration measures the amount of biomass in a mixture of water and biomass, where a high concentration indicates a greater amount of biomass in a relatively low amount of water. Therefore, at high feed concentrations or at less water content in feedstock mixture, reforming, hydrolysis, and water–gas shift reactions diminish as per Le Chatelier’s principle, which decreases the hydrogen yield at high feedstock concentrations [46]. An increase in carbon content (C) improved the hydrogen yield up to a certain extent; however, a further increase in carbon content did not improve hydrogen yield. Thus, biomass with high carbon content is only beneficial up to a certain extent. 

These results are in agreement with the reported literature of SCWG of lignocellulosic biomass, where the temperature is the most important parameter for the SCWG process, followed by reaction time and feedstock concentration, while reaction pressure is the least influential reaction parameter for SCWG of lignocellulosic biomass [47]. Among biomass properties, hydrogen content (H), ash content (ash), and carbon content (C) were the most dominant features, which indicates biomass with high hydrogen content and a moderate amount of ash and carbon content is recommended for high hydrogen yield. Overall, biomass properties had a high feature importance of 52.91% compared to feature importance of 47.09% of SCWG reaction process parameters for the prediction of hydrogen yield. This highlights the importance of screening most suitable lignocellulosic biomass feedstocks to achieve high hydrogen yield. Thus, although optimization of SCWG conditions is necessary for improving hydrogen yield, more attention should be paid to the selection of suitable lignocellulose biomass for holistic optimization of the SCWG process for maximization of hydrogen yield. 

Feature analysis based on CatBoost-PSO for methane yield showed that the carbon content (C) of the biomass was the most dominant feature, with its highest feature importance of 24.85%, followed by (17.65%) temperature and (8.78%) volatile matter (VM) of biomass (Figure 8). An increase in the carbon content of the biomass increased the methane yield. This is due to the fact that carbon molecules in SCWG product gas are facilitated by the carbon content of the biomass. An increase in reaction temperature also increased the methane yield, which is due to the hydrogenation and methanation of produced CO and CO_2_ at high reaction temperatures, which increased the methane yield at high reaction temperatures [48]. Similarly, an increase in volatile matter (VM) of biomass enhanced methane yield due to the ease of gasification of volatile matters in SCWG. These volatile matters represent the alcohols, ketones, aldehydes, and organic acids. These are the intermediates of the gasification in SCW, which are easily converted into gaseous products such as methane, H_2_, and CO_2_ [13]. Therefore, an increase in the volatile matter of biomass increased methane yield. Similar to hydrogen yield, biomass properties had cumulative feature importance of 63.19% compared to 36.81% feature importance of SCWG reaction conditions. 

For CO gas yield, feature analysis revealed that the ash content (ash), volatile matter (VM), temperature, time, and concentration were the most influential features, with feature importance of 16.93, 15.35, 15.05, 14.27, and 11.08%, respectively (Figure 9). Low to moderate ash content had a negative impact, which is due to the fact that even though AAEMs in ash content enhances reforming reactions, due to enhancement of water–gas shift reactions, most of the produced CO is consumed for hydrogen production. Only at a really high ash content, where the water–gas shift reaction attains equilibrium and an increased content of AAEMs, the water–gas shift reaction is no longer enhanced, leading to the increase in CO yield. This was also observed in hydrogen yield where really high ash content actually decreased the hydrogen yield. Similarly, an increase in volatile matter decreased the CO yield as high volatile matter promotes the further conversion of CO gas into methane and hydrogen via water–gas shift, methanation, and hydrogenation reactions. Similarly, an increase in temperature and time also decreases CO yield, as at high reaction temperature, water–gas shift reactions dominate and consume the CO. CO yield is high at short reaction times as a short reaction time does not allow sufficient time for further conversion of produced CO by consecutive water–gas shift and methanation reactions, which are enabled at longer reaction times. This led to the decrement in CO yield at longer reaction times. However, an increase in feedstock concentration increased the CO yield. This is due to diminished activity of water–gas shift, methanation, and hydrogenation reactions at high feedstock concentrations, which result in unutilized CO gas and thus increases the yield of CO gas at higher feedstock concentrations [49]. 

Similar to methane yield, carbon content (C) of the biomass was the most dominant feature for prediction of CO_2_ gas yield, having feature importance of 29.73%, which is followed by (12.64%) temperature, (9.14%) volatile matter (VM), and (8.84%) time (Figure 10). This is because most of the carbon content comes from the biomass itself, which resulted in its highest feature importance for prediction of CO_2_ yield. Thus, an increase in carbon content of biomass increased the CO_2_ gas yield. An increase in temperature increases the conversion of CO to CO_2_ and hydrogen by enhancing the water–gas shift reaction. Similarly, an increase in volatile matter of biomass favors the production of gaseous products due to the ease of gasification of volatile matter resulting in an increase in yield of CO_2_ [50].

An increase in time also allows sufficient time for further conversion of produced CO gas by enhanced water–gas shift reactions at longer reaction time, which increases the CO_2_ gas yield. 

It can be observed that even though temperature has a high influence among SCWG process features on gas yields of the SCWG process, biomass properties as a whole have feature importance of 52.91, 63.19, 57.54, and 68.37% compared to 47.09, 36.81, 42.46, and 31.63% feature importance of SCWG process parameters for prediction of H_2_, CH_4_, CO, and CO_2_ gas yields, respectively. Thus, biomass characteristic plays a key role in the SCWG degradation mechanism of lignocellulosic biomass, which influences the gas distribution of the SCWG process. The characteristics of biomass should be considered while optimizing SCWG process parameters. These biomass properties and the SCWG process also have interactive effects during the gasification of lignocellulosic biomass in SCW. Hence, study of the interactive effects of these input features on gas yields of SCWG is important to understand the degradation mechanism of lignocellulosic biomass in SCWG. 

### 3.4. Two-Way SHAP Analysis

**Figure 11:**
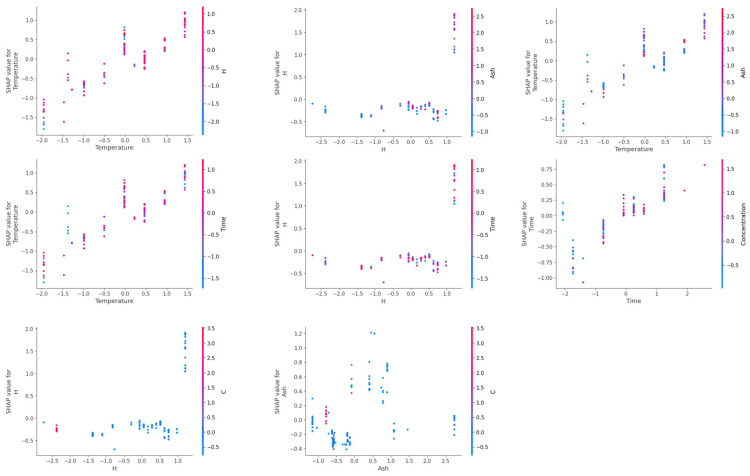
SHAP two-way plot of interactive behavior of the most important input features on prediction of H_2_ yield in the PSO-optimized XGB model.

**Figure 12:**
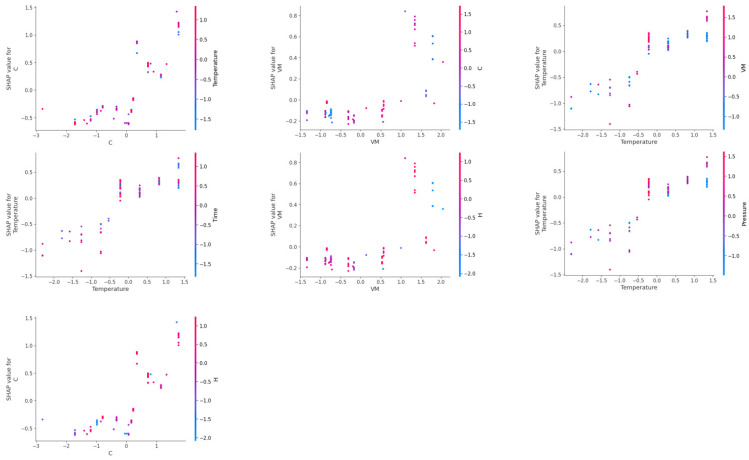
SHAP two-way plot of interactive behavior of the most important input features on prediction of CH_4_ yield in the PSO-optimized CatBoost model.

**Figure 13:**
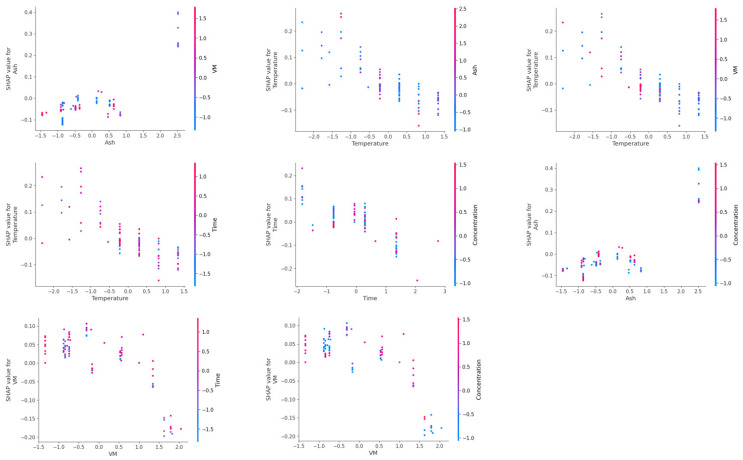
SHAP two-way plot of interactive behavior of the most important input features on prediction of CO yield in the PSO-optimized CatBoost model.

**Figure 14:**
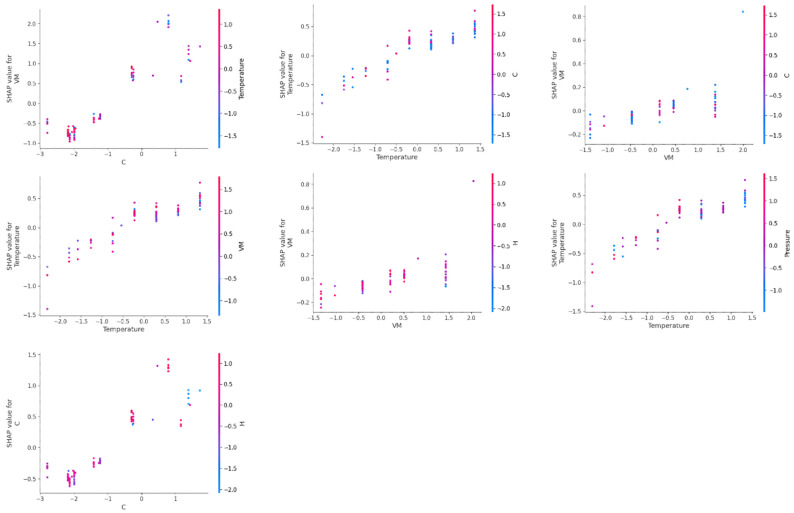
SHAP two-way plot of interactive behavior of the most important input features on prediction of CO_2_ yield in the PSO-optimized CatBoost model.

SHAP dependency plots for investigating the influence of interactive effects of the most dominant input features for the prediction of gas yields are presented in Figure 11, Figure 12, Figure 13 and Figure 14. In a SHAP two-way dependency plot, the *x*-axis shows the value of feature 1 and the primary *y*-axis represents the effect as the function of the SHAP values of the target variable (gas yields). The effect of feature 2 is represented using the secondary *y*-axis and values are represented using a gradient. This helps to visualize the interactive effects of the two input variables on the SHAP values of the gas yields. 

From Figure 11, it can be observed that the input features had interactive effects on the prediction of hydrogen yield. An increase in temperature for high hydrogen content containing biomass resulted in the highest SHAP values for hydrogen yield. High SHAP values for hydrogen yield can also be achieved for moderate hydrogen containing biomass at high reaction temperatures. However, high hydrogen content at low reaction temperatures does not necessarily translate into high hydrogen yield. Similarly, modest ash content helped to achieve high hydrogen yields at high reaction temperatures. However, low reaction temperature even at optimum Ash content does not result in high hydrogen yield. For hydrogen content (H) and ash content of biomass, these features did not show much interaction at a low hydrogen content and low ash content of biomass. Only at an optimum ash content of biomass did an increase in hydrogen content of biomass result in the highest hydrogen yield.

Similarly, for time and hydrogen content, the highest hydrogen yield was obtained at highest hydrogen content and longer reaction time. However, at shorter reaction times and low high hydrogen content, these features did now show much interaction for hydrogen yield. This indicates that for efficient conversion of the hydrogen content of biomass into hydrogen gas during gasification, higher reaction temperature, longer reaction time, and optimum amount of ash content are required. Reaction temperature and reaction time showed interactive behavior, where highest hydrogen yields were obtained at high reaction temperature and longer reaction time. However, a comparable hydrogen yield can also be obtained even at short reaction times at high reaction temperatures. Similarly, reaction time and concentration also showed interactive behavior, and the highest hydrogen yield was obtained at low feedstock concentration at longer reaction times. A high hydrogen yield was obtained even at moderate to high concentrations at longer reaction times. 

For prediction of methane yield, carbon content (C) and temperature showed high interactive behavior (Figure 12). The highest SHAP values for methane yield were obtained at high carbon content and high reaction temperatures. Strong interactive behavior was observed at moderate to high values of carbon content and at high reaction temperatures. Volatile matter (VM) and carbon content also showed high interactive behavior, and biomass having high volatile matter usually had moderate to high carbon content, which resulted in the highest methane yield. Temperature and VM of biomass also had strong interactive behavior, and high methane yield was obtained at a high reaction temperature and high amount of volatile matter. However, high SHAP values of methane yield were also observed at moderate VM at high temperatures or, also at moderate temperatures for biomass having high VM content. Temperature and time had interactive behavior for SHAP values of methane yield at high temperature and longer reaction time. Similarly, hydrogen and carbon content of biomass had an interactive effect on methane yield at high carbon and high hydrogen content, which resulted in the highest SHAP values of methane yield. 

Interestingly, ash content and volatile matter (VM) of biomass had interactive behavior on SHAP values of CO yield; only at high ash content and moderate volatile matter were the highest SHAP values of CO gas yield observed (Figure 13). However, temperature had strong interactive behavior with ash content, volatile matter, and time. High SHAP values of CO yield were obtained at low temperatures and low ash content of biomass. Similarly, low temperature and moderate values of volatile matter resulted in the highest SHAP values of CO yield. However, high values of CO yields were obtained at low reaction times at low temperatures. Time also had strong interactive behavior with concentration and volatile matter of biomass for CO yield, where high SHAP values of CO yield were observed at short reaction times and high concentrations. However, low values of volatile matter at short reaction times resulted in high SHAP values for CO yield. Volatile matter and concentration themselves also had a strong interactive influence on CO yield, where high SHAP values of CO yield were obtained at low volatile matter and high feedstock concentration. 

Two-way SHAP analysis for prediction of CO_2_ yield showed that the carbon content (C) of biomass and temperature had a strong interactive influence on SHAP values of CO_2_ yield. The highest values of CO_2_ yield were observed at high carbon content and high temperatures (Figure 14). Similarly, carbon content of biomass also had interactive behavior with volatile matter on CO_2_ yield, where an increase in volatile matter and carbon content increased the SHAP values of CO_2_ yield. Temperature and volatile matter also had interactive effects at high values of temperature and volatile matter, where an increase in volatile matter at high reaction temperature increased the SHAP values of CO_2_ yield. However, high comparable values of SHAP values were also observed at moderate volatile matter at high temperatures. 

Temperature and time also had a strong interactive influence on CO_2_ yield. Longer reaction time and high temperature had the highest SHAP values of CO_2_ yield. Hydrogen content also demonstrated strong interactive behavior with volatile matter and carbon content of biomass on SHAP values of CO_2_ yield. High volatile matter and high hydrogen content showed the highest values of CO_2_ yield. This is due to the relationship between volatile matter and hydrogen content of biomass, as volatile matter of biomass represents high quantities of organic acids, hydrocarbons, alcohols, aldehydes, and ketones. These compounds usually have higher amounts of hydrogen atoms; thus, an increase in volatile matter also represents an increase in hydrogen content, which had a positive interactive influence on the SHAP values of CO_2_ yield. High values of hydrogen content and carbon content also resulted in high values of CO_2_ yield.

Thus, the degradation of lignocellulosic biomass follows a complex reaction mechanism, and input variables such as SCWG reaction conditions and biomass properties have an interactive influence during SCWG of lignocellulosic biomass. These interactive influences of input features have an effect on the product distribution and individual gas yields of the SCWG process. Two-way SHAP analysis highlighted the strong interactive influence of the most dominant features on yields of H_2_, CH_4_, CO, and CO_2_. This shows that the optimization of SCWG of lignocellulosic biomass is a complex process and requires careful simultaneous tuning of various parameters to maximize the hydrogen yield of the SCWG process. 

Thus, this study presented a novel and comprehensive application of machine learning models for SCWG of lignocellulosic biomass to elucidate the interactive effects of input features and their complex relationship with gas yields. Utilization of only lab-scale batch reactor data helped to better capture the relationships between input variables and gas yields with minimum influence of other unaccounted-for variables. However, it also resulted in limited scope of the prediction models only to a lab-scale batch reactor. Nevertheless, the main objective of this study was to understand the complex degradation behavior of lignocellulosic biomass during SCWG and interactive effects of input variables on gas yields. This study presented a groundwork for comprehensive optimization of SCWG reaction conditions and selection of suitable biomass, especially at industrial scale, to maximize hydrogen gas yields with a high degree of certainty. This will foster efforts being made for commercialization of SCWG at an industrial scale. 

## 4. Conclusions

In conclusion, this study showed the successful implementation of machine learning models for prediction of gas yields of SCWG of lignocellulosic biomass. Among the eight screened machine learning model for eight prediction of gas yield of SCWG, boosting ensemble tree models such as XGB and CatBoost models demonstrated superior prediction power. For prediction of H_2_ yield, PSO-optimized XGB showed eight highest test R^2^ of 0.84, whereas, for prediction of CH_4_, CO, and CO_2_ gas yields, PSO-optimized CatBoost showed highest test R^2^ of 0.83, 0.94, and 0.92, respectively. This is due to the use of series of multiple simple decision tree models by boosting ensemble tree models, which improves upon the preceding tree and prevents the overfitting of the dataset. Even though both GA and PSO are heuristic algorithms, the use of the PSO optimizer was more effective compared to the GA optimizer in hyperparameter tuning of machine learning models for improving their prediction power. This is due to the difference in the search mechanisms of these algorithms. 

Feature analysis based on PSO-optimized XGB showed that temperature was the most dominant feature for prediction of H_2_ yield, with its highest feature importance of 21.93%. For prediction of CH_4_, CO, and CO_2_ gas yields by the PSO-optimized CatBoost model, carbon content (C), ash content (ash), and carbon content (C) were the most dominant features, with feature importance of 24.85, 16.93, and 29.73%, respectively. Among SCWG reaction conditions and biomass characteristics, biomass characteristics were most dominant as a whole, with cumulative feature importance of 52.91, 63.19, 57.54, and 68.37% for prediction of H_2_, CH_4_, CO, and CO_2_ gas yields from SCWG of lignocellulosic biomass, respectively. SHAP two-way analysis revealed strong interactive behavior of input features for the prediction of H_2_, CH_4_, CO, and CO_2_ gas yields and their effect on the SCWG reaction mechanism. This also confirmed the complex and non-linear nature of the SCWG process. Furthermore, the high importance of biomass characteristics highlights the importance of the selection of suitable feedstocks and understanding the interactive behavior of components of hydrogenous biomass for efficient conversion of lignocellulosic biomass into high hydrogen yield. This work has laid the framework for a comprehensive optimization of the SCWG process, which can be a key asset for the commercialization of the SCWG process at an industrial scale.

## Figures and Tables

**Table 1:** Parameters of the genetic algorithm (GA) for hyperparameter tuning of machine learning models.

Parameters	Numbers
Generation (generations)	50
Crossover probability (crossover_prob)	0.8
Population size (population_size)	100
Mutation probability (mutation_prob)	0.2

**Table 2:** Parameters of the particle swarm optimization (PSO) algorithm for hyperparameter tuning of machine learning models.

Parameters	Numbers
Number of particles (n_particles)	30
Number of iterations (max_iter)	10
Inertial weight (alpha)	0.5
Personal attachment (beta)	1.5
Global attraction (gamma)	1.5

**Table 3:** Optimized hyperparameters of machine learning models by GA and PSO optimizers and the search range for the prediction of hydrogen yield.

Hyperparameter	Range	Unoptimized	GA	PSO
Random forest (RF)				
n_estimators	10–500	100	187	10
max_depth	1–50	NaN *	13	41
min_samples_split	2–10	2	3	2
min_samples_leaf	1–10	1	1	1
extreme gradient boosting (XGB)				
learning_rate	0.01–0.5	NaN	0.402014	0.268485
n_estimators	50–500	NaN	408	500
max_depth	3–10	NaN	9	5
min_child_weight	1–7	NaN	5	1
gamma	0–0.5	NaN	0.141899	0.267668
subsample	0.5–1	NaN	0.989653	0.715084
colsample_bytree	0.5–1	NaN	0.975411	0.500000
Decision tree (DT)				
max_depth	1–50	NaN	49	50
min_samples_split	2–50	NaN	8	4
min_samples_leaf	1–50	NaN	7	1
Support vector machine (SVM)				
C	0.1–1000	1	462.771600	74.618075
epsilon	0.01–1	0.1	0.424860	0.141974
gamma	0.1–1		0.035418	0.045675
Categorical boosting regressor (CatBoost)				
learning_rate	0.01–0.5	0.03	0.252239	0.439795
depth	4–10	6	7	5
l2_leaf_reg	1–10	3	7.786251	9.244647
Artificial neural network (ANN)				
learning_rate_init	0.0001–0.1	0.001	0.010889	0.035365
hidden_layer_sizes	5–100	100	81.428966	36.580111
activation_function	identity, logistic, tanh, relu	relu	tanh	logistic
Gaussian process regression (GPR)				
sigma	0.0001–55	1	0.315774	0.050060
kernel_function	RBF *, Matern	RBF	RBF	RBF

* NaN: Not a Number; RBF: Radial Basis Function.

## Data Availability

Data available upon request.
